# The discovery of PCSK9 as a pivotal point in the prevention of cardiovascular disease

**DOI:** 10.1016/j.ajpc.2025.101314

**Published:** 2025-09-22

**Authors:** Alexander C. Razavi, Michael D. Shapiro

**Affiliations:** a Emory Center for Heart Disease Prevention, Emory University School of Medicine, Atlanta, GA, USA; b Center for Prevention of Cardiovascular Disease, Section on Cardiovascular Medicine, Wake Forest University School of Medicine, Winston-Salem, NC, USA

**Keywords:** PCSK9 protein, PCSK9 inhibitors, Prevention, Cardiovascular disease

## Abstract

Proprotein convertase subtilisin/kexin type 9 (PCSK9) is an important component in the regulation of low-density lipoprotein-cholesterol (LDL-C) metabolism, discovered more than two decades ago. The discovery of PCSK9 and subsequent development of PCSK9 inhibitors (PCSK9i) have helped usher a new era of non-statin lipid-lowering therapy for atherosclerotic cardiovascular disease (ASCVD) risk reduction. In addition to individuals with clinical ASCVD, there have been evolving recommendations for the clinical use of PCSK9i to extend to individuals with familial hypercholesterolemia as well as high-risk primary prevention patients, including those with advanced subclinical atherosclerosis. Beyond the initial development of PCSK9 monoclonal antibodies (mAb), this class of therapies has expanded to include several different modes of administration that are currently being studied for efficacy and safety, including small interfering RNA (siRNA), adnectins, oral, and potentially even CRISPR-based methods. Such scientific advancement and enthusiasm have been moderated by public health challenges involving cost and access of therapy, which we hope will continue to improve in an era emphasizing earlier and greater utilization of combination lipid-lowering therapy. In this review, we will summarize PCSK9 biology, followed by an assessment of completed and ongoing randomized controlled trials involving PCSK9i. This will then be followed by a review of current clinical recommendations for the utilization of PCSK9i, future directions, and concluded with clinical and public health impact. Through this process, we hope to highlight the integral role of PCSK9i in the prevention of ASCVD.

**Figure:**
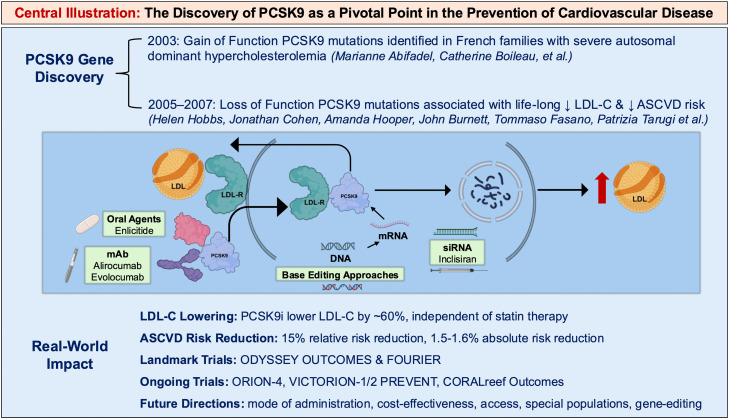
Central Illustration. The Discovery of PCSK9 as a Pivotal Point in the Prevention of Cardiovascular Disease. Legend: PCSK9 binds to the LDL receptor on hepatocytes, promoting receptor degradation in lysosomes and raising plasma LDL-C levels. This figure illustrates multiple therapeutic strategies targeting PCSK9 at different stages: monoclonal antibodies (evolocumab, alirocumab) or macrocyclic peptides (enlicitide) bind circulating PCSK9, siRNA inhibits PCSK9 mRNA translation, and base editing introduces a single nucleotide change in the PCSK9 gene that generates a premature stop codon, resulting in a loss-of-function allele and lifelong reduction of functional PCSK9 protein. Together, these interventions increase LDL receptor recycling, lowering circulating LDL-C.

## 1 Discovery of PCSK9

The proprotein convertases are a group of nine secretory serine proteases that share commonality in their ability to hydrolyze peptide bonds to promote cellular processes across a wide range of biological functions [1]. Proprotein convertase subtilisin/kexin type 9 (PCSK9) is an important component in the regulation of low-density lipoprotein-cholesterol (LDL-C) metabolism, discovered more than two decades ago. The initial discovery occurred in 2003, as Abifadel, Boileau, and collaborators identified a relationship between gain-of-function (GOF) mutations in the *PCSK9* gene and autosomal dominant hypercholesterolemia when studying French families with hypercholesterolemia [2]. Dr. Helen Hobbs and colleagues subsequent work would later identify loss-of-function (LOF) variants in *PCSK9* associated with lifelong lower LDL-C (heterozygous LOF ∼60mg/dL, homozygous LOF ∼15 mg/dL) and lower coronary heart disease (CHD) risk [[3], [4], [5], [6]]. Such evidence would ultimately lead to the development of PCSK9 inhibitors (PCSK9i), including humanized monoclonal antibodies (mAb), which have been show to lower LDL-C by ∼60 % and confer a 15 % relative reduction (1.5–1.6 % absolute risk reduction) in atherosclerotic cardiovascular disease (ASCVD) risk among individuals already on statin therapy [7,8]. More recently, small-interfering ribonucleic acid (siRNA), oral, and adnectin preparations of PCSK9i are under study in Phase 2 and/or Phase 3 randomized controlled trials (RCT) [9].

Overall, the discovery of PCSK9 and subsequent development of PCSK9i have helped usher a new era of non-statin lipid-lowering therapy for ASCVD risk reduction [10]. In this review, we will summarize PCSK9 biology, followed by an assessment of completed and ongoing RCT involving PCSK9i. This will then be followed by a review of current clinical recommendations for the utilization of PCSK9i, future directions, and concluded with clinical and public health impact. Through this process, we hope to highlight the integral role of PCSK9i in the prevention of ASCVD.

## 2 PCSK9 structure and biology

**Fig. 1:**
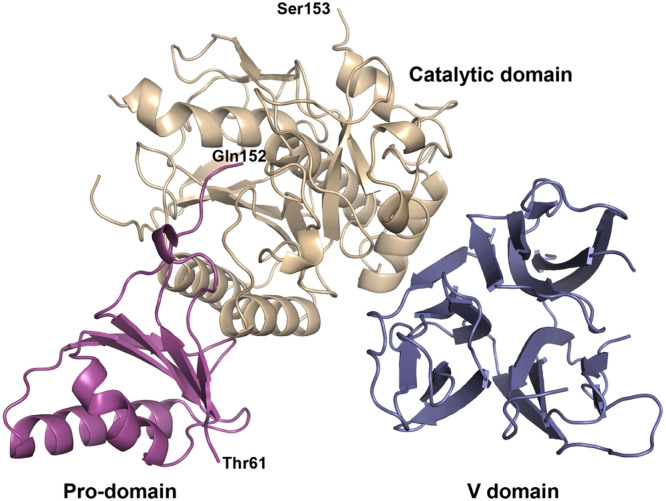
Protein structure of PCSK9. The Pro-domain (magenta) is autocatalytically cleaved and facilitates protein folding. The catalytic domain (wheat) contains protease activity that is conserved among all proprotein convertases and can bind to the LDL-receptor. The V domain (also known as the C-terminal domain) is most important for PCSK9 interaction with the LDL-receptor, directing the PCSK9-LDL-receptor complex for degradation by endosomes and lysosomes. Thr61 is the N-terminus and Gln152 is the C-terminus of the Pro-domain. Ser153 is the N-terminus of the Catalytic domain. Figure reproduced from: Piper DE et al. The Crystal Structure of PCSK9: A Regulator of Plasma LDL-Cholesterol [12].

The *PCSK9* gene is found on chromosome 1 and can produce two different isoforms via alternative splicing of 15 exons within the gene [11]. Major tissues that express PCSK9 include the liver, small intestine, kidney, and skin. The PCSK9 protein is initially synthesized as a proprotein in the endoplasmic reticulum, undergoes autocatalytic cleavage, and is then transported to the Golgi apparatus for secretion into circulation [10]. The protein structure of PCSK9 is comprised of four components (Fig. 1). The catalytic domain, regulated by the N-terminal prodomain, is directly linked to the function of PCSK9 - facilitating binding and ultimately degradation of the LDL-receptor (LDLR) [12].

**Fig. 2:**
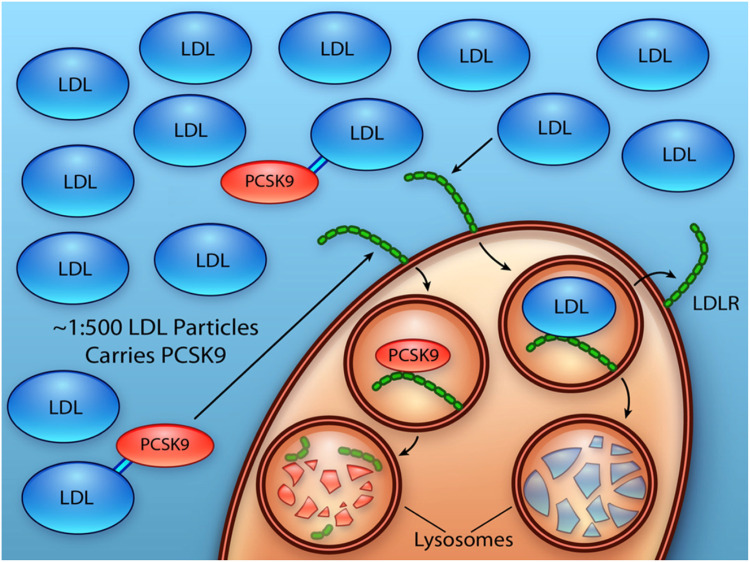
Interaction between PCSK9 and LDL-Receptor. Each LDLR is recycled approximately every 10 min with a lifecycle of nearly one day, permitting one LDLR to facilitate uptake of many LDL particles (∼100–150). This process is regulated by PCSK9, as PCSK9 enters the cell via the LDLR, facilitating its internalization within coated vesicles and shuttling LDLR to the lysosome for degradation. In circulation, approximately 50–60% of PCSK9 is bound to LDL particles such that there is one PCSK9 bound to every 500–1000 LDL particles. Figure reproduced from: Shapiro MD, Tavori H, Fazio S. PCSK9: From Basic Science Discoveries to Clinical Trials [10].

The biology of the LDLR was first discovered by Nobel Laureates, Brown and Goldstein in 1970 [13]. In brief, they demonstrated that LDL was internalized by cells via receptor-mediated endocytosis driven by binding of apolipoprotein-B (ApoB) on each LDL particle to LDLR. Each LDLR is then recycled approximately every 10 min with a lifecycle of nearly one day, permitting one LDLR to facilitate uptake of many LDL particles (∼100–150) [10]. This process is regulated by PCSK9, as PCSK9 enters the cell via the LDLR, facilitating its internalization within coated vesicles and shuttling LDLR to the lysosome for degradation [14] (Fig. 2). In circulation, approximately 50–60 % of PCSK9 is bound to LDL particles such that there is one PCSK9 bound to every 500–1000 LDL particles [15,16]. While up to 60 % of PCSK9 is found on LDL particles, most LDL particles are not harboring PCSK9 as it is a relatively low abundance plasma protein. Overall, there is reciprocal regulation between PCSK9 and LDLR – as PCSK9 is a ligand for LDLR, helping to facilitate its exit from the circulation, and PCSK9 in turn facilitates the internalization of LDLR [10].

Given its role in facilitating LDLR degradation, PCSK9 activity ultimately raises circulating cholesterol, which have led to questions regarding the evolutionary role of PCSK9. Several hypotheses suggest that PCSK9 evolved to combat intracellular entry of specific viruses and bacteria; however, such a current role for the protein is questionable [17]. In fact, LOF PCSK9 variants in humans are associated with improved survival in sepsis due to increased availability of LDLR to facilitate pathogen removal and a decreased inflammatory cytokine response [18]. Among 60 individuals hospitalized for severe coronavirus-19 (COVID-19) infection, evolocumab versus placebo reduced the risk death or intubation (23 % versus 53 %) as well as interleukin-6 levels (−56 % versus 21 %) over 30-days [19]. However, meta-analysis of twenty randomized controlled trials demonstrates that PCSK9i are not significantly associated with a reduced or increased risk of sepsis or severe infections as serious adverse events [20]. Future RCTs that are adequately powered and designed to assess sepsis and severe infections as primary outcomes may help provide further clarity and insights on the role of PCSK9i modulating the risk of infection.

Following the discovery of PCSK9, there was considerable interest in evaluating circulating PCSK9 as a potential prognostic biomarker. Many studies have demonstrated a direct linear association between higher levels of PCSK9 and subclinical atherosclerosis across different modalities, including carotid intima-media thickness on ultrasound [21], coronary artery calcium (CAC) on non-contrast cardiac computed tomography [22,23], as well as coronary atherosclerotic lesions as detected on intravascular ultrasound [24]. However, not all prior work has observed an association between PCSK9 and subclinical atherosclerosis [25], which has mirrored inconsistent findings for PCSK9 predicting incident and recurrent ASCVD events [[26], [27], [28]]. In the largest study to identify an independent association between PCSK9 and ASCVD among more than 4200 persons without prior CHD or stroke, individuals in the fourth versus bottom quartile of PCSK9 had a 48 % higher relative risk of incident ASCVD, though the addition of PCSK9 to traditional risk factors did not improve the C-statistic or net reclassification index [29]. Similar inconsistent results have been observed in populations with clinical ASCVD [30,31], such that PCSK9 has only been associated with outcomes in subgroups of secondary prevention and/or has not been significantly associated with recurrent events after adjusting for traditional risk factors.

Despite the integral role of PCSK9 in regulating LDL-C, its relatively modest association with ASCVD outcomes is likely related to its biology in circulation. As discussed earlier, only approximately 40–50 % of circulating PCSK9 is bound to ApoB particles with the remaining proportion involved in separate biological pathways [10]. Thus, the inconsistent association between plasma PCSK9 levels and ASCVD risk may be explained by the fact that only PCSK9 bound to apoB-containing particles (such as LDL and Lp(a)) is likely to be relevant to cardiovascular outcomes. It is conceivable that assays capable of distinguishing between free and apoB-bound PCSK9 may offer a more informative assessment of its prognostic value in ASCVD risk stratification

## 3 Clinical trials of PCSK9 inhibition

### 3.1 Monoclonal antibodies

The first humanized monoclonal antibodies (mAb) targeting PCSK9 to receive Food and Drug Administration (FDA) approval were evolocumab and alirocumab in 2015 ^32^. Of note, prior Phase 2 trials demonstrated that humanized mAb led to a 60 % reduction in LDL-C among individuals on statin therapy, with no attenuation in LDL-C lowering over time [33,34]. The most common adverse event in such trials was a small increase in injection site reactions.

The first Phase 3 outcome trials involving PCSK9 mAb therapy were published more than a half-decade ago (Table 1). In 2017, the FOURIER (Further Cardiovascular Outcomes Research with PCSK9 Inhibition in Subjects with Elevated Risk) trial demonstrated that nearly 28,000 persons with chronic, stable CHD with LDL-*C* ≥ 70 mg/dL on statin therapy who were randomized to evolocumab versus placebo experienced a 1.5 % absolute risk reduction in composite ASCVD over 2.2 years (number needed to treat [NNT]=67)[7]. One year later, the ODYSSEY Outcomes (Evaluation of Cardiovascular Outcomes After an Acute Coronary Syndrome During Treatment with Alirocumab) showed that alirocumab led to a 1.6 % absolute risk reduction (NNT=63) over 2.8 years among nearly 19,000 individuals who had experienced acute coronary syndrome within the last 12 months and had inadequately controlled cholesterol on statins (LDL-*C* ≥ 70, non-HDL-*C* ≥ 100, or apolipoprotein-B [ApoB] ≥80 mg/dL) [8].

**Table 1:** Completed and ongoing Phase 3 cardiovascular outcome trials evaluating PCSK9 inhibitors.

Trial Name	Patient Population, (N)	PCSK9 Inhibitor	Follow-Up	Outcomes & Results
**Monoclonal Antibody**				
FOURIER	Stable ASCVD, (24,081)	Evolocumab (Repatha)	2.2 years	• 15 % relative risk reduction (1.5 % absolute risk reduction) in cardiovascular death, MI, hospitalization for unstable angina, coronary revascularization
ODYSSEY Outcomes	Recent ACS, (18,924)	Alirocumab (Praluent)	2.8 years	• 15 % relative risk reduction (1.6 % absolute risk reduction) in CHD, nonfatal MI, fatal or nonfatal ischemic stroke, or unstable angina requiring hospitalization
**siRNA**				
ORION-4	Stable ASCVD, (∼16,124)	Inclisiran (Leqvio)	∼5 years	• Projected completion by 2026 • Outcome: composite outcome of CHD death, MI, fatal or nonfatal ischemic stroke, or urgent revascularization procedure
VICTORION-1 PREVENT	High-risk primary prevention defined by: coronary artery stenosis 20–69 % (20–49 % in left main), CAC ≥100, 10-year ASCVD risk ≥20 % or 7.5–19 % with ≥2 risk-enhancing factors, (∼14,013)	Inclisiran (Leqvio)	∼6.25 years	• Projected completion by 2029 • Outcome: composite of 4-point MACE: cardiovascular death, non-fatal MI, non-fatal ischemic stroke, and urgent coronary revascularization
VICTORION-2 PREVENT	Stable ASCVD (including PAD), (∼17,004)	Inclisiran (Leqvio)	∼6 years	• Projected completion by 2027 • Outcome: composite of 3-point MACE: cardiovascular death, non-fatal MI, and non-fatal ischemic stroke.
**Oral**				
CORALreef Outcomes	Stable ASCVD or high-risk primary prevention, (∼14,550)	Enlicitide Decanoate	∼6 years	• Projected completion by 2029 • Outcome: composite of cardiovascular death, non-fatal MI, non-fatal ischemic stroke, acute limb ischemia or major amputation, and urgent arterial revascularization

ACS=acute coronary syndrome, ASCVD=atherosclerotic cardiovascular disease, CHD=coronary heart disease, MACE=major adverse cardiovascular events, MI=myocardial infarction, PAD=peripheral arterial disease.

Given that the median duration of follow-up in the FOURIER and ODYSSEY Outcomes trials were 2–3 years, longer-term follow-up studies involving PCSK9 inhibition have been performed. Among the 27,564 participants in the FOURIER trial, 6635 individuals enrolled in an open-label extension study (FOURIER-OLE) which yielded a maximum duration of follow-up of 8.4 years (median 5 years) [35]. The absolute risk reduction in composite ASCVD with evolocumab versus placebo was 0.6 % in the open-label extension of the FOURIER trial. In FOURIER-OLE, the median achieved LDL-C was 30 mg/dL, which was not associated with any adverse events, including diabetes, hemorrhagic stroke, or neurocognitive events [35]. Although both groups received evolocumab in the FOURIER-OLE follow-up period, those randomized to placebo in the parent trial had persistently higher ASCVD event rates compared to those randomized to evolocumab in the parent trial, suggesting that achieving earlier and longer LDL-C control is most optimal [35]. Neurocognitive safety has additionally been demonstrated in the EBBINGHAUS (Evaluating PCSK9 Binding Antibody Influence on Cognitive Health in High Cardiovascular Risk Subjects), which was a substudy of FOURIER and demonstrated no statistically significant change in executive function among individuals randomized to evolocumab who achieved a median LDL-C of 35 mg/dL over 5.1 years follow-up [36]. Real-world effectiveness and safety data involving PCSK9 mAb have mirrored RCT findings [37].

### 3.2 Small interfering RNA

Beyond mAb, there has been an ongoing interest in evaluating the efficacy of PCSK9 inhibition via small-interfering ribonucleic acid (siRNA) which may be administered less frequently. Both PCSK9 mAb and siRNA therapies are administered subcutaneously but differ fundamentally in mechanism. The mAbs act extracellularly by binding circulating PCSK9, whereas siRNA works intracellularly by interfering with translation of PCSK9 mRNA. Because circulating PCSK9 levels are relatively low, neutralizing it with mAbs effectively inhibits both existing and newly secreted protein. In contrast, siRNA therapy uses N-acetylgalactosamine (GalNAc) conjugation to enable efficient targeting to hepatocytes, where most PCSK9 is synthesized. This liver-specific delivery allows for lower doses, improved potency, and reduced systemic side effects.

Prior work has demonstrated that inclisiran, a siRNA targeting hepatic expression of PCSK9, leads to a 53 % reduction in LDL-C at 180 days follow-up after two doses of inclisiran spaced 3 months apart [38]. Longer term follow-up data from the ORION-10 and ORION-11 trials demonstrated a sustained 50 % reduction in LDL-C over approximately one-year of follow-up with inclisiran dosed every 6 months [39]. Nearly all individuals in these trials had clinical ASCVD and approximately 90 % were taking statins, with two-thirds on high-intensity therapy. Subgroup analyses in ORION-11 including more than 200 high-risk participants without clinical ASCVD has demonstrated similar reductions in LDL-C [40].

Such work has ultimately led to ongoing Phase 3 outcome trials evaluating the efficacy of inclisiran for reducing the risk of ASCVD events (Table 1). The ORION-4 trial was the first of such trial started in 2018 and enrollment has been affected by the COVID-19 pandemic. Results from ORION-4 are estimated to be released in 2026 and seeks to assess whether inclisiran reduces recurrent events over a median follow-up of 5 years among men >40 years old and women >55 years old with clinical ASCVD [41]. Similar ongoing outcome trials evaluating inclisiran include the VICTORION-1 PREVENT (high risk, no clinical ASCVD)[42] and VICTORION-2 PREVENT (stable, clinical ASCVD)[43]. The VICTORION-INCEPTION trial is evaluating the efficacy of inclisiran in the post-acute coronary syndrome setting. Preliminary results have demonstrated that inclisiran reduces LDL-C by 45 %, with approximately two-thirds achieving LDL-*C* < 70 mg/dL within one-year of follow-up [44]. Lastly, the VICTORION-INITIATE trial is evaluating whether an “inclisiran first” strategy for individuals with LDL-*C* ≥ 70 mg/dL and clinical ASCVD will lead to a reduction in ASCVD events compared to usual care. Thus far, results from VICTORION-INITIATE demonstrate superiority in those randomized to inclisiran compared to usual care for achieving LDL-*C* < 70 (82 % versus 22 %) and <55 mg/dL (72 % versus 9 %)[45]. Usual care in this trial was according to physician discretion, which resulted in most patients receiving statins alone (73 %) and less than 3 % utilization of combination lipid-lowering therapy.

The inclisiran dosing regimen for all such trials is based on prior work that has demonstrated an approximate 50 % LDL-C lowering with inclisiran dosed at baseline, 3 months, and then every 6 months thereafter. Thus, siRNA inhibition of PCSK9 offers the benefit of less frequent dosing intervals, though subcutaneous injection of inclisiran is given in office due to the higher injection volume compared to mAb evolocumab or alirocumab, the latter which can be administered by patients at home. There have been no direct head-to-head comparisons of siRNA versus mAb inhibition, and both approaches are highly efficacious for LDL-C lowering over at least one-year follow-up. However, PCSK9 mAb have demonstrated an incrementally higher LDL-C lowering efficacy compared to siRNA therapy (∼60 % versus ∼50 %)[46].

### 3.3 Current clinical recommendations

Recommendations and guidance regarding the utilization of PCSK9i have evolved since the 2018 Multi-Society Cholesterol Guideline (Table 2). In 2018, United States recommendations for the utilization of PCSK9 mAb were constructed leveraging initial short-term follow-up data from the ODYSSEY and FOURIER trials. As such, the 2018 Multi-Society guideline recommended the utilization of ezetimibe prior to PCSK9 mAb as the initial non-statin therapy for individuals requiring additional LDL-C lowering [47]. Specific patient groups noted were individuals on maximally tolerated statin therapy and ezetimibe with clinical ASCVD at very-high risk (recurrent events or one prior event and multiple high-risk conditions) when LDL-*C* ≥ 70 mg/dL or non-HDL-*C* ≥ 100 mg/dL (Class 2a) or heterozygous familial hypercholesterolemia (HeFH) with LDL-*C* ≥ 100 mg/dL (Class 2b)[47].

**Table 2:** Current United States guidelines and expert consensus recommendations for the utilization of PCSK9i therapy.

Guideline / Consensus Document	Recommendation
2018 Multi-Society Cholesterol Guideline	• Clinical ASCVD with very-high risk who have LDL-*C* ≥ 70 mg/dL or non-HDL-*C* ≥ 100 mg/dL despite maximally tolerated statin and ezetimibe therapy (Class 2a) • LDL-*C* ≥ 190 mg/dL who have not achieved LDL-*C* < 100 mg/dL despite maximally tolerated statin and ezetimibe therapy (Class 2b)
2022 ACC Expert Consensus Decision Pathway on Non-Statin Therapy	• Clinical ASCVD with very-high risk who have LDL-*C* ≥ 70 mg/dL or non-HDL-*C* ≥ 100 mg/dL despite maximally tolerated statin therapy (consider *concurrently* with ezetimibe) • Clinical ASCVD not at very-high risk who have LDL-*C* ≥ 70 mg/dL (consider *after* ezetimibe therapy) • Familial hypercholesterolemia or LDL-*C* ≥ 190 mg/dL who have not achieved LDL-*C* < 100 mg/dL or <50 % reduction despite maximally tolerated statin therapy (consider *concurrently* ezetimibe therapy) • CAC ≥1000 with LDL-*C* ≥ 70 mg/dL on maximally tolerated statin therapy (consider *after* ezetimibe therapy) • Statin intolerant patients • *consider inclisiran for patients who are unable to self-inject PCSK9 mAb
2024 Primary Prevention of Stroke Guideline	• Adults without clinical ASCVD with LDL-*C* ≥ 190 mg/dL, 10-year risk ≥20 % or 7.5–19 % with at least one risk-enhancer who cannot reach LDL-C goal on statin therapy (Class 2b)
2025 Guideline for the Management of Acute Coronary Syndrome	• In patients with ACS who are already on maximally tolerated statin therapy with LDL-C 55 to 69 mg/dL, adding a non-statin lipid-lowering therapy (including PCSK9i) is reasonable to reduce risk of MACE (Class 2a)

The 2022 American College of Cardiology Expert Consensus Decision Pathway (ECDP) on Non-Statin Therapies provide an expanded potential use of PCSK9i for ASCVD risk reduction, incorporating longer-term follow-up data from the ODYSSEY and FOURIER trials as well as randomized controlled trial evidence with inclisiran. One of the main updates within the 2022 ACC ECDP was that PCSK9 mAb were recommended to be considered concurrently with ezetimibe as the initial choice of non-statin therapy for individuals with very-high risk ASCVD and clinical ASCVD with LDL-*C* ≥ 190 mg/dL or familial hypercholesterolemia that require additional LDL-C lowering [32]. For such individuals unable to self-inject PCSK9 mAb, guidance was also provided to consider in-office administration of inclisiran. Additional potential PCSK9 benefit groups outlined in the 2022 ACC ECDP on non-statin therapies include, clinical ASCVD not at very-high risk or those with CAC ≥1000 with LDL-*C* ≥ 70 mg/dL on maximally tolerated statin therapy and ezetimibe [32]. Most recently, the 2025 Guideline for the Management of Patients with Acute Coronary Syndrome provides a Class 2a recommendation for the addition of non-statin therapy, including PCSK9i therapy, for individuals with acute coronary syndrome and LDL-C to 55 to 69 mg/dL to reduce ASCVD risk [48].

We anticipate that future iterations of guidelines will continue to highlight the integral role of PCSK9i for ASCVD risk reduction. While there are several key developing areas, notable considerations include the evolving use of PCSK9i in high-risk primary prevention and advanced subclinical atherosclerosis, utilization of inclisiran, as well as the incorporation of updated cost effectiveness data. The VESALIUS-CV (Effect of Evolocumab in Patients at High Cardiovascular Risk Without Prior Myocardial Infarction or Stroke) trial assessing the effect of LDL-C with evolocumab on MACE among individuals without prior myocardial infarction or stroke who have LDL-*C* ≥ 90, non-HDL-*C* ≥ 120, or ApoB ≥80 mg/dL with significant vascular disease (coronary heart disease, cerebrovascular disease, peripheral arterial disease), diabetes with retinopathy or nephropathy or ≥10 years, and/or high-risk primary prevention conditions, including coronary artery calcium ≥100[49]. Of note, the 2024 Primary Prevention of Stroke Guideline provided a Class 2b recommendation for the use of PCSK9, citing that future studies are required to assess whether there is clear benefit extended to individuals without prior ASCVD [50,51].

## 4 Future directions

### 4.1 Mode of administration

There are several future directions involving PCKS9i, including mode of administration, cost-effectiveness and access, as well as special patient populations (Table 3). Additional trials Enlicitide is an oral PCSK9i farthest along in development and is being evaluated in the CORALreef HeFH (A Study of Enlicitide Decanoate (Oral PCSK9 Inhibitor) in Adults with Heterozygous Familial Hypercholesterolemia)[52] and CORALreef AddOn (A Study to Evaluate the Efficacy and Safety of Enlicitide Decanoate (Oral PCSK9 Inhibitor) Compared with Ezetimibe or Bempedoic Acid or Ezetimibe and Bempedoic Acid in Adults with Hypercholesterolemia). Initial results from the CORALreef trials demonstrate significant reductions in LDL with oral PCSK9i, enlicitide, when compared to placebo in heterozygous familial hypercholesterolemia (FH) and when compared to ezetimibe and bemepedoic acid in those with hypercholesterolemia [53,54]. Beyond enlicitide, the PURSUIT (A Study to Assess the Efficacy, Safety and Tolerability of Different Doses of AZD0780 in Patients with Dyslipidemia) was a Phase 2 RCT evaluating the efficacy of oral PCSK9i among individuals with hypercholesterolemia on moderate or high-intensity statin therapy with LDL-*C* ≥ 70 mg/dL. After 12 weeks, there was a stepwise higher LDL-C lowering with higher doses of oral PCSK9 therapy with 1 (−35 %), 3 (−38 %), 10 (−45 %), and 30 mg (−50 %) versus placebo, without any increase in adverse events [55].

**Table 3:** Future directions involving PCSK9 inhibition.

Area	Progress / Future Directions
Mode of Administration	Oral Therapy, Adectin, CRISPR
Cost-Effectiveness and Access	Annual and monthly cost have decrease by ∼60 % since initial FDA-approval, though still remain expensive and cost-prohibitive for many patients
Special Patient Populations	High-risk primary prevention, elevated Lp(a), mild-to-moderate aortic stenosis, statin intolerant

Beyond oral administration, additional efforts have been dedicated towards further improving the delivery mechanism of extracellular PCSK9i via adnectins. Adnectins are 15-fold smaller than monoclonal antibodies and are easily producible, synthetic binding proteins developed from the 10th type 3 domain of human fibronectin [56]. Adnectins bind to albumin in serum, increasing the half-life and allowing for monthly injection of the adnectin-based PCSK9i, lerodalcibep [57,58]. In addition, injection volume of adnectin-based lerodalcibep is smaller when compared to traditional monoclonal antibodies, evolocumab and alirocumab. Monthly injection of 300 mg lerodalcibep versus placebo led to a 65 % reduction over 24 weeks among individuals with heterozygous FH in the Liberate-HeFH trial [59]. Nearly all individuals in the trial were on statin therapy (88 %) and there was no increase in adverse events beyond injection site reactions with lerodalcibep, similar to PCSK9 mAb therapy [59].

In addition to adnectin delivery mechanisms, permanent base-pair editing within the *PCSK9* gene via clustered regularly interspaced short palindromic repeats (CRISPR) may also be a possibility. VERVE-102 is a clinical stage in-vivo CRISPR base-editing therapy that changes one DNA base in the *PCSK9* gene [60]. Thus far, VERVE-102 versus placebo has led to a maximum 80 % reduction in circulating PCSK9 protein and a 62 % reduction in LDL-C after single dosing [60]. The Heart-2 trial is an ongoing Phase 1b in humans to evaluate the optimal dose and safety of VERVE-102 among individuals ages 18–65 with heterozygous familial hypercholesterolemia or premature coronary artery disease [61]. Similar to siRNA PCSK9 therapy, VERVE-102 CRISPR base-editing therapy is via a GalNAc delivery system for hepatocyte uptake.

Though it has been approximately one decade since FDA approval of PCSK9i therapy, cost-effectiveness and access remain significant barriers. Initially, the average annual cost of PCSK9i therapy was close to $14,000, though more recent data demonstrate that this price has decreased by approximately 60 % to $5850[62]. Despite these cost reductions, evidence regarding real-world utilization of PCSK9i suggests a very-low prescription rate among eligible individuals (∼1 %)[63]. In a survey of potential barriers to PCSK9i prescription, insurer processes, inadequate documentation, and administrative burden have been cited as leading obstacles [64]. We anticipate that the cost of therapy will continue to decrease as the market of PCSK9i agents continues to expand. Additionally, the benefit of earlier and necessary combination lipid-lowering therapy to achieve LDL-C goals will continue to be stressed in upcoming consensus statements and potential guideline updates.

In addition to the guideline-indicated groups that benefit from PCSK9i therapy, there are certain special patient populations to highlight. Much of this evidence comes from post-hoc analyses of PCSK9i RCTs. First, individuals with elevated Lp(a) have been shown to derive a greater magnitude of benefit from PCSK9i compared to those with lower Lp(a) values. In a post-hoc analysis of the FOURIER trial, participants with Lp(a) ≥median (37 nmol/L) had a larger absolute risk reduction with evolocumab compared to those with Lp(a) <median (2.5 % versus 1 %)[65]. Such differences led to large differences in the number needed to treat to prevent one recurrent ASCVD event with evolocumab for individuals with Lp(a) ≥median versus <median (40 versus 105)[64]. Similar findings have been observed in post-hoc analyses of the ODYSSEY Outcomes trial, including individuals with recent acute coronary syndrome [66,67]. Meta-analyses of RCTs, demonstrate that PCSK9 mAb lower Lp(a) by approximately 25 %[68].

The modest Lp(a) lowering properties of PCSK9i led to an exploratory analysis in FOURIER to assess whether evolocumab was associated with a reduced risk of aortic stenosis (AS). Importantly Lp(a) is among the strongest, causal risk factors for AS. In FOURIER, evolocumab versus placebo was associated with a non-significant 44 % lower relative risk of incident AS over 2.2 years follow-up [69]. When stratifying the association according to follow-up time, evolocumab was significantly associated with a lower risk of AS after the first year (HR=0.48, 95 % CI: 0.25–0.93) but not within the first year of follow-up (HR=1.09, 95 % CI: 0.48–2.47)[69]. There are several ongoing Phase 2 trials in this space, evaluating whether PCSK9i versus placebo may reduce the progression of AS [70,71].

## 5 Conclusions, clinical and public health impact

In summary, the discovery of PCSK9 and generation of PCSK9i have had a transformative role in preventive cardiology. Clinically, they have expanded our armamentarium of lipid-lowering therapy beyond statins, which is especially important given the very-low proportion of individuals with clinical ASCVD on statin therapy that achieve LDL-C goals. In addition to individuals with clinical ASCVD, there have been evolving recommendations for the use of PCSK9i clinically to extend to individuals with FH as well as high-risk primary prevention patients, including those with advanced subclinical atherosclerosis. Beyond the initial development of PCSK9 mAb, this class of therapies has expanded to include several different modes of administration that are currently being studied for efficacy and safety, including siRNA, adnectin, oral, and potentially even CRISPR-based methods. Such scientific advancement and enthusiasm have been moderated by public health challenges involving cost and access of therapy, which we hope will continue to improve in an era emphasizing earlier and greater utilization of combination lipid-lowering therapy. We anticipate that PCSK9i will occupy a key role in the future of preventive cardiology and ASCVD risk reduction.

## CRediT authorship contribution statement

**Alexander C. Razavi:** Writing – review & editing. **Michael D. Shapiro:** Writing – review & editing, Writing – original draft, Conceptualization.

## Declaration of competing interest

None.
